# Lessons Learned From COVID-19 Contact Tracing During a Public Health Emergency: A Prospective Implementation Study

**DOI:** 10.3389/fpubh.2021.721952

**Published:** 2021-08-20

**Authors:** Tyler Shelby, Christopher Schenck, Brian Weeks, Justin Goodwin, Rachel Hennein, Xin Zhou, Donna Spiegelman, Lauretta E. Grau, Linda Niccolai, Maritza Bond, J. Lucian Davis

**Affiliations:** ^1^Department of Epidemiology of Microbial Diseases, Yale School of Public Health, New Haven, CT, United States; ^2^Yale School of Medicine, New Haven, CT, United States; ^3^New Haven Health Department, New Haven, CT, United States; ^4^Department of Biostatistics, Yale School of Public Health, New Haven, CT, United States; ^5^Center for Methods in Implementation and Prevention Science, Yale School of Public Health, New Haven, CT, United States; ^6^Pulmonary, Critical Care and Sleep Medicine Section, Yale School of Medicine, New Haven, CT, United States

**Keywords:** COVID-19, contact tracing, implementation science, health equity, infectious disease outbreak

## Abstract

**Background:** Contact tracing is a core element of the public health response to emerging infectious diseases including COVID-19. Better understanding the implementation context of contact tracing for pandemics, including individual- and systems-level predictors of success, is critical to preparing for future epidemics.

**Methods:** We carried out a prospective implementation study of an emergency volunteer contact tracing program established in New Haven, Connecticut between April 4 and May 19, 2020. We assessed the yield and timeliness of case and contact outreach in reference to CDC benchmarks, and identified individual and programmatic predictors of successful implementation using multivariable regression models. We synthesized our findings using the RE-AIM implementation framework.

**Results:** Case investigators interviewed only 826 (48%) of 1,705 cases and were unable to reach 545 (32%) because of incomplete information and 334 (20%) who missed or declined repeated outreach calls. Contact notifiers reached just 687 (28%) of 2,437 reported contacts, and were unable to reach 1,597 (66%) with incomplete information and 153 (6%) who missed or declined repeated outreach calls. The median time-to-case-interview was 5 days and time-to-contact-notification 8 days. However, among notified contacts with complete time data, 457 (71%) were reached within 6 days of exposure. The least likely groups to be interviewed were elderly (adjusted relative risk, aRR 0.74, 95% CI 0.61–0.89, *p* = 0.012, vs. young adult) and Black/African-American cases (aRR 0.88, 95% CI 0.80–0.97, pairwise *p* = 0.01, vs. Hispanic/Latinx). However, ties between cases and their contacts strongly influenced contact notification success (Intraclass Correlation Coefficient (ICC) 0.60). Surging caseloads and high volunteer turnover (case investigator *n* = 144, median time from sign-up to retirement from program was 4 weeks) required the program to supplement the volunteer workforce with paid public health nurses.

**Conclusions:** An emergency volunteer-run contact tracing program fell short of CDC benchmarks for time and yield, largely due to difficulty collecting the information required for outreach to cases and contacts. To improve uptake, contact tracing programs must professionalize the workforce; better integrate testing and tracing services; capitalize on positive social influences between cases and contacts; and address racial and age-related disparities through enhanced community engagement.

## Introduction

Coronavirus Disease 2019 (COVID-19) emerged in late 2019 and rapidly spread throughout the world with dramatic effects on health systems and societies (1). Contact tracing and other non-pharmaceutical interventions have assumed critical importance for limiting the spread of SARS-CoV-2 (2) and will remain important in protecting unvaccinated populations and responding to breakthrough transmission from variant strains. Contact tracing is a complex intervention that involves isolating and investigating cases while eliciting, quarantining, and monitoring their close contacts. Although, contact tracing is effective for mitigating many communicable diseases including sexually transmitted infections (3) and tuberculosis (4), it must be tailored to the clinical features and transmission dynamics of the causative pathogen, as well as the local epidemiological context and resources. In East Asia, e.g., contact tracing was rapidly and effectively adapted for COVID-19 thanks to early and massive political and financial investments, informed by prior experiences with Severe Acute Respiratory Syndrome (SARS) (5) and Middle East Respiratory Syndrome (MERS) (6) and largely receptive societies. When combined with other preventive measures including physical distancing, universal masking, and digital tracking, contact tracing for COVID-19 has been shown to reduce the effective reproductive number (R_e_) (7), secondary attack rates (8) and case fatality rates (9, 10) and to contain outbreaks and generalized epidemics in diverse settings (10–12). Nevertheless, contact tracing has not proven effective everywhere (13, 14), and many have questioned its overall usefulness in the recent pandemic (15).

Given these uncertainties, a better understanding of the implementation of contact tracing is critical to learning from the COVID-19 pandemic and preparing for the future. Modeling suggests that the effectiveness of contact tracing depends on the speed and efficiency with which cases are isolated and contacts quarantined (16). Target benchmarks proposed by the U.S. Centers for Disease Control and Prevention (CDC) include successfully investigating ≥60% of cases and placing their contacts in quarantine within 6 days of exposure (17). While media outlets have covered implementation of contact tracing extensively, very few scientific reports have evaluated the implementation fidelity or context or explored individual or health system risk factors for dropping out of contact tracing (18–21). Therefore, we sought to evaluate measures and determinants of implementation for a COVID-19 contact tracing program rapidly established in New Haven, Connecticut in early 2020.

## Methods

### Setting and Contact Tracing Procedures

New Haven, a racially and ethnically diverse city of 130,250 residents (33% Black/African-American, 31% Hispanic/Latinx, 30% White, 5% Asian) (22) confirmed its first COVID-19 cases in mid-March 2020. Working together, the New Haven Health Department (NHHD) and the Yale School of Public Health launched an emergency contact tracing program for the City of New Haven on April 4 using the city's existing emergency management software (Veoci, New Haven, CT). Students, faculty, and staff in the graduate health sciences at Yale University were recruited into a volunteer workforce of 151 case investigators and 36 contact notifiers (both henceforth labeled “contact tracers”), as previously described (23). In early April, 40 public health nurses from the NHHD were added to the case investigation team.

Each day, the city's lead epidemiologist sent a list of newly reported COVID-19 cases to volunteer leaders, who then assigned them to case investigators. Case investigators were instructed to telephone cases within 24 h and identify close contacts, defined as those with whom the case had spent ≥15 min within a six-foot radius during the infectious period (16). If a case did not answer, investigators were instructed to leave a voicemail message and try again daily for 3 days.

Contact names, phone numbers and exposure dates (henceforth termed “outreach information”) were securely emailed to volunteer coordinators for distribution to contact notifiers. Notifiers telephoned contacts to inform them about their exposure to COVID-19, and counsel them to self-monitor for symptoms, seek testing if symptomatic, and self-quarantine for 14 days after the last exposure date. Contacts were not called if missing outreach information or if reported >14 days after exposure.

### Study Design and Participants

We evaluated each of the processes involved implementing contact tracing using quantitative data recorded for the NHHD. We included all COVID-19 cases with a specimen collection date between April 4 and May 19 (when Connecticut began reopening businesses), except cases residing in congregate settings (e.g., nursing homes). We included all close contacts of eligible cases.

### Measurements and Outcomes

We obtained demographic data for cases and contacts and dates of testing and tracing events from local registries. We defined six key steps of contact tracing (Supplementary Figure 1), beginning with collection of the diagnostic specimen from the case. These included [1] reporting cases to the NHHD, [2] telephoning cases, [3] interviewing cases, [4] reporting contacts, [5] telephoning contacts, and [6] notifying contacts. We produced indicators of yield and timeliness for each step and used the CDC target benchmarks as specified above (24). To quantify the availability of human resources, we used shift records to estimate the weekly person-hours contributed by public health nurses and volunteers.

### Analysis Plan

We presented characteristics of telephoned cases and contacts using proportions for dichotomous variables and medians with quartiles for continuous variables. We calculated yield indicators as stepwise and cumulative proportions and presented them using flow diagrams and a descriptive cascade. We calculated timeliness indicators as the cumulative time from specimen collection to completion of key processes and presented them using violin plots. We excluded observations with missing or non-sensical time values (e.g., notification date preceding outreach date).

In addition, we constructed three multivariable models using generalized estimating equations (GEE) (25), employing a log link function to obtain multivariable-adjusted relative risks (aRR) for each covariate. Each model evaluated the associations between case, contact, and program characteristics and indicators of success at one of three points in the cascade: [A] completion of the case interview for all cases telephoned, [B] collection of outreach information for all contacts, and [C] completion of notification for all contacts telephoned. We included all case, contact, and program covariates in the models, as long as there were at least 10 outcomes per variable (26). We grouped categorical responses with fewer than 10 outcomes and used largest categories as reference groups. We used multiple imputation (27) to account for missing covariate data and reported the results obtained using the imputed data. We included a variable for calendar week of case registration or contact identification to assess temporal trends, as well as a variable for programmatic capacity (ratio of the total contact tracer-hours available each week to incident cases or contacts to be telephoned each week). We estimated unadjusted intraclass coefficients (ICCs) using GEE (28) to account for correlation among outcomes of cases assigned to the same investigator, and outcomes of contacts elicited by the same investigator, reported by the same case, or called by the same notifier. For additional details on these analyses, see Supplementary Text Methods.

Last, we compared the weekly person-hours available to the case investigation team (supply), and incident cases to be telephoned (demand) over time, estimating a 1-h average duration for each case investigation (29), and plotted volunteer retention over time. We estimated the effect of time-since-volunteer-sign-up on weekly hours volunteered per individual with a multivariable GEE model, adjusted for calendar week of sign-up. A lack of data on characteristics of individual volunteers prevented us from adjusting for additional characteristics.

We synthesized findings using the RE-AIM framework, a widely used approach to evaluating implementation. According to RE-AIM, the ***E****ffectiveness* of an intervention depends on a series of conditional processes, including uptake by participants (***R****each*) and implementers (***A****doption*), delivery (***I****mplementation*), and sustainability (***M****aintenance*) (30). We characterized the *reach* of contact tracing based on indicators of yield and predictors of completion; its *implementation* based on timeliness; and its *adoption* and *maintenance* based on availability, demand, and retention metrics for contact tracers. Sample size was based on convenience, and statistical significance assessed in reference to a *p*-value < 0.05. Analyses were carried out in STATA version 16 (College Station, TX), Microsoft Excel (Redmond, WA) and SAS 9.4 (Cary, NC).

### Human Subjects

The Yale Human Subjects Committee approved the study protocol and waived the requirement for informed consent on grounds of minimal risk.

## Results

### Study Sample and Cascade Yields

**Figure 1:**
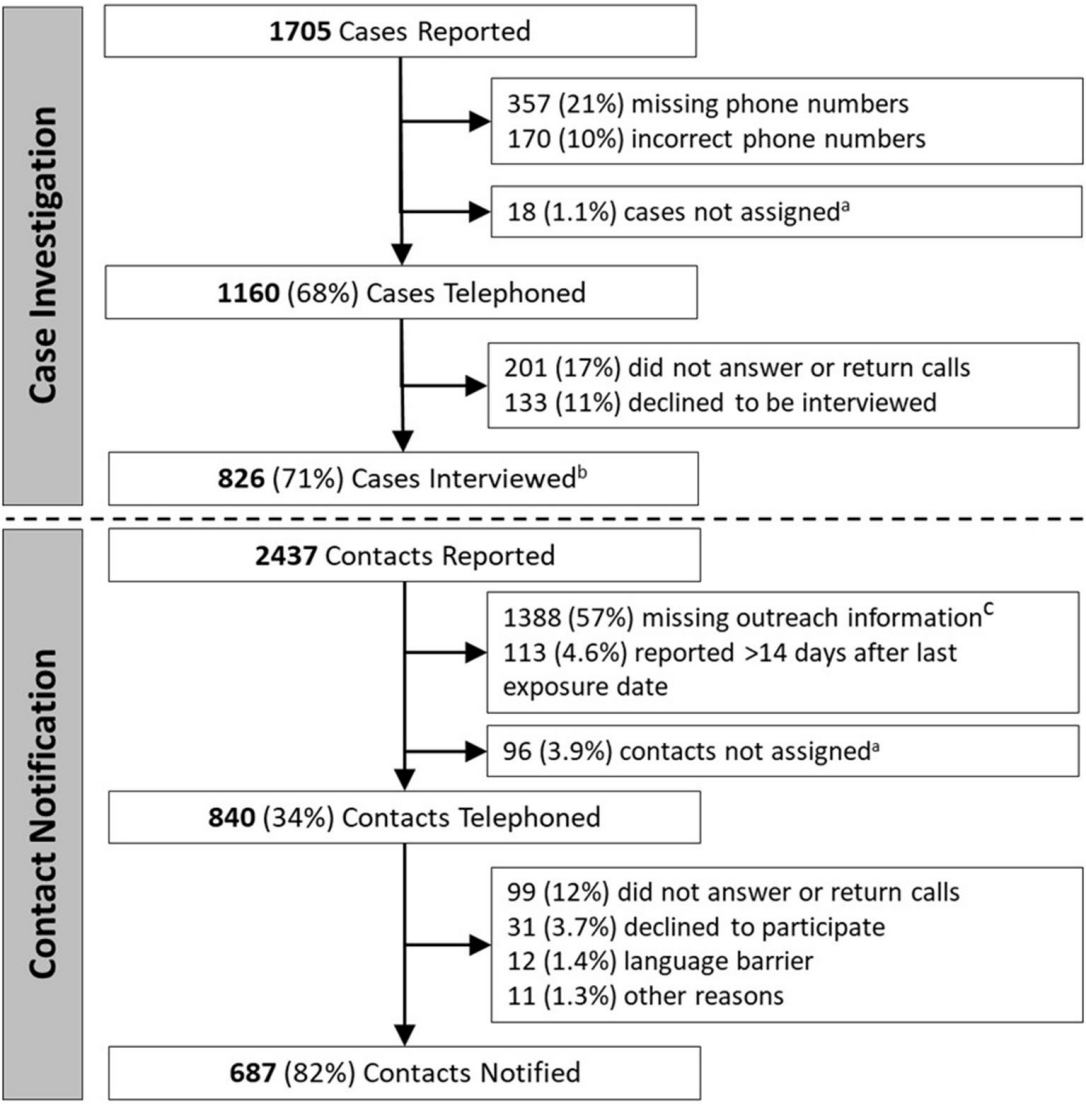
Stepwise yield of key steps of case investigation and contact notification. ^a^Cases or contacts were occasionally not successfully assigned, such as when volunteers were unable to receive assignments or when contact data was not transferred between case investigation and contact notification teams. ^b^737/826 (89%) interviewed cases reported one or more contacts; ^c^972/2,437 (40%) reported contacts were missing/incorrect phone number, 683/2,437 (28%) were missing last exposure date, and 341/2,437 (14%) were missing name.

There were 1,705 COVID-19 cases reported to the NHHD during the evaluation period (Figure 1). Of these, 527 (31%) had missing (357, 21%), or incorrect (170, 10%) phone numbers, while 18 (1.1%) were not successfully assigned to case investigators. Among the remaining 1,160 (68%) cases telephoned, 201 (17%) did not answer or return calls, and 133 (11%) answered but declined to participate. The remaining 826 (71%) cases were interviewed, and of these, 737 (89%) reported one or more contacts. Characteristics of the 1,160 cases telephoned are shown in Table 1.

**Table 1:** Baseline Characteristics of Cases Telephoned^a^ and Contacts Telephoned^a^.

**Characteristic**	***n* (%)^**b**^**
**Cases (** ***n*** **=** **1160)**	
Age^c^, median years (Q1–Q3)	41 (28–54)
<18	64 (5.6)
18–35	384 (34)
36–50	329 (29)
51–65	244 (22)
>65	115 (10)
Female^d^	644 (57)
Race/ethnicity^d^	
Hispanic/latinx	537 (54)
Black/African-American	322 (32)
Caucasian/White	106 (11)
Other	30 (3.0)
**Contacts (** ***n*** **=** **840)**	
Age^f^, median years (Q1–Q3)	32 (18–48)
<18	170 (24)
18–35	240 (33)
36–50	153 (21)
50–65	111 (15)
>65	44 (6.1)
Female	510 (61)
Household contact of case	695 (83)
Relationship to case	
Family member	722 (86)
Social contact	91 (11)
Work contact	27 (3.2)

*Q1, quartile 1; Q3, quartile 3.*

a *Baseline characteristics were not available for all cases reported or for all contacts reported.*

b *Unless otherwise specified;*

c *24 missing;*

d *37 missing;*

e *165 missing;*

f *122 missing*.

Interviewed cases reported a total of 2,437 contacts (a median of 2 contacts per case) (Figure 1). Of these, 1,388 (57%) lacked outreach information, including 972 (40%) with missing/incorrect phone numbers, 683 (28%) with missing exposure dates, and 341 (14%) with missing names. Another 113 (4.6%) were identified >14 days after last exposure date, and 96 (3.9%) were not successfully assigned to volunteers. Of the remaining 840 (34%) who were telephoned, 687 (82%) were successfully notified, while 99 (12%) did not answer or return calls, 31 (3.7%) answered but declined to participate, 12 (1.4%) were not reached due to language barriers, and 11 (1.3%) were not reached for other reasons. The characteristics of the 840 contacts telephoned are shown in Table 1.

Ultimately, investigators interviewed 48% of all cases, with 32% lost before being telephoned and 20% lost before being interviewed (Supplementary Figure 2). Of all contacts, 28% were notified, with 66% lost before being telephoned, and 6% lost before being notified.

### Timeliness

**Figure 2:**
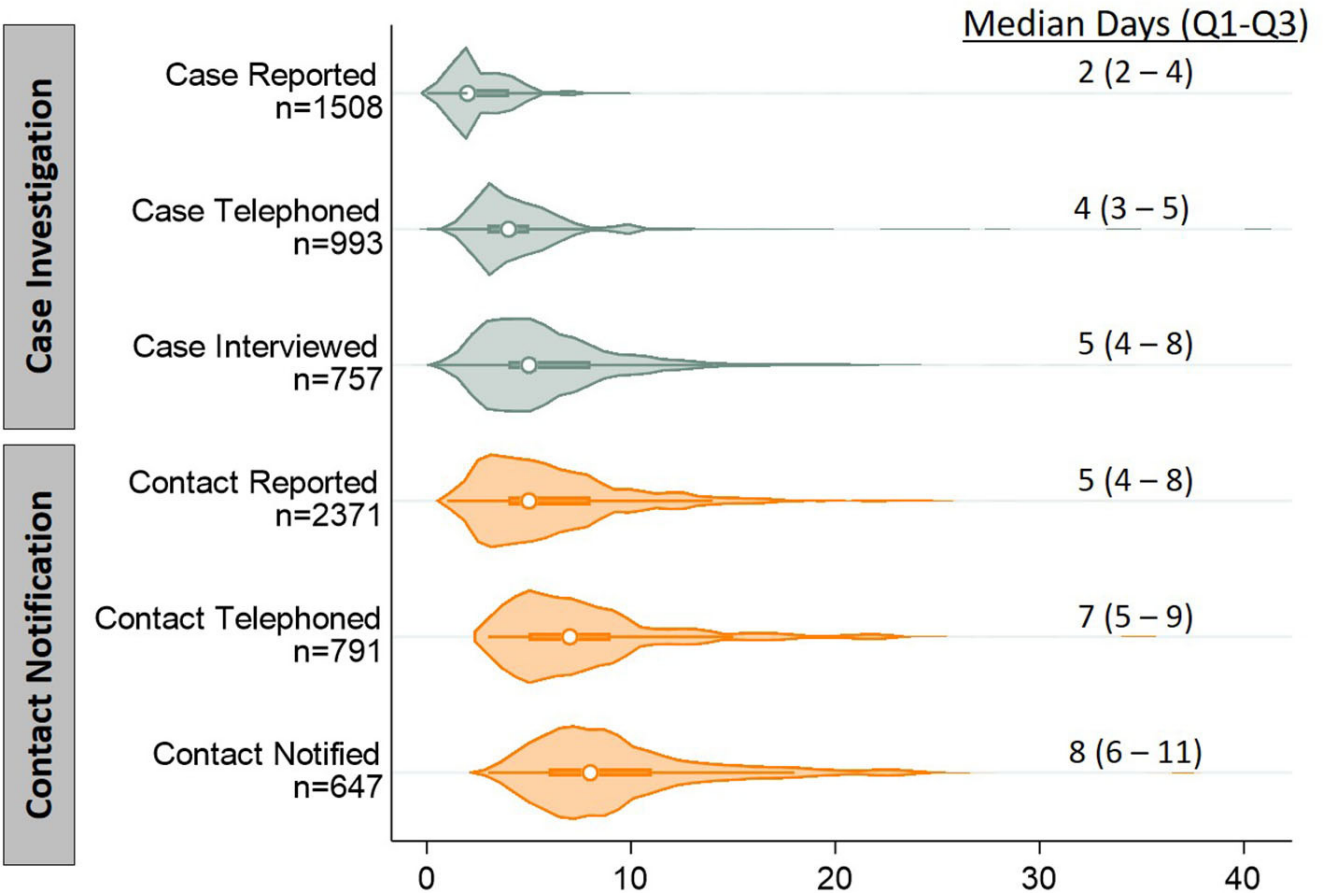
Violin plots depicting distributions of timeliness indicators for key steps of contact tracing, in days. Timeliness indicators were calculated as the cumulative time from specimen collection from a case to completion of each of the six steps of contact tracing (subdivided into case investigation and contact notification). Each indicator includes only participants who completed that step and had the initiation and completion times recorded. The displayed n's differ from those presented in Figure 1 because of missing time data (either the case's report date or any subsequent event date). We also excluded 29 contact observations with non-sensical time values (e.g., notification date preceding outreach date). Violin plots show distributions as a shaded, smoothed kernel density estimator; inside the distribution plot, medians are plotted as an open circle and the upper and lower quartile range is plotted as a bolded line.

The median time from case specimen collection to case reporting to NHHD was 2 days (Quartile 1 (Q1) - Quartile 3 (Q3): 2–4); to telephoning cases, 4 days (Q1–Q3: 3–5); and to case interview, 5 days (Q1–Q3: 4–8) (Figure 2). The median time to contact reporting was 5 days (Q1–Q3: 4–8); to telephoning contacts, 7 days (Q1–Q3: 5–9); and to contact notification, 8 days (Q1–Q3: 6–11). Among the 648 notified contacts with valid dates recorded for most recent exposure and notification, 457 (71%) were notified within 6 days of their exposure.

### Factors Associated With Successful Implementation

Among 1,160 cases telephoned, several factors were significantly associated with interview completion (Supplementary Table 1). The probability of being interviewed was lower for the elderly (aRR for >65 years old vs. young adult (18–35 years): 0.74, 95% CI 0.61–0.89, *p* = 0.012). Although race as a whole was not a significant predictor, Black/African American cases were significantly less likely than Hispanic/Latinx cases to be interviewed (aRR: 0.88, 95% CI 0.80–0.97, pairwise *p* = 0.01). Furthermore, the probability of success decreased by 3% for each calendar week following initiation of the program (aRR: 0.97, 95% CI 0.94–0.99, *p* = 0.020). Success rates did not vary substantially among interviewers (ICC = 0.002).

Among the 2,437 contacts reported, the probability of collecting all required outreach information was lower for contacts reported by cases aged 36–50 years old (aRR 0.83, 95% CI 0.73–0.93, *p* = 0.008, vs. young adult cases). Probability of collecting outreach information was also lower for contacts <18 years vs. young adult (aRR 0.63, 95% CI 0.54–0.72, *p* < 0.001), non-household vs. household contacts (aRR 0.88, 95% CI 0.77–1.00, *p* = 0.0495), social vs. family contacts (aRR 0.77, 95% CI 0.65–0.91, *p* < 0.001) and work vs. family contacts (aRR 0.57, 95% CI 0.44–0.74, *p* < 0.001) (Supplementary Table 2). Success rates varied by case interviewer (ICC = 0.21), suggesting that the way questions are asked may influence outcomes. Success rates also varied by case cluster (ICC = 0.45), indicating that cases who provide outreach information for any individual contact are more likely to provide it for other contacts they report.

For the 840 contacts telephoned, the probability of notification was influenced by the ratio of contact notifiers to contacts (aRR 1.43, 95% CI 1.04–1.95, *p* = 0.026) (Supplementary Table 3). Notification rates varied only modestly by contact notifier (ICC = 0.14) but varied more substantially by case cluster (ICC = 0.60), suggesting that ties between cases and their contacts may influence the success of contact outreach.

### Volunteer Case Investigator Adoption and Maintenance

**Figure 3:**
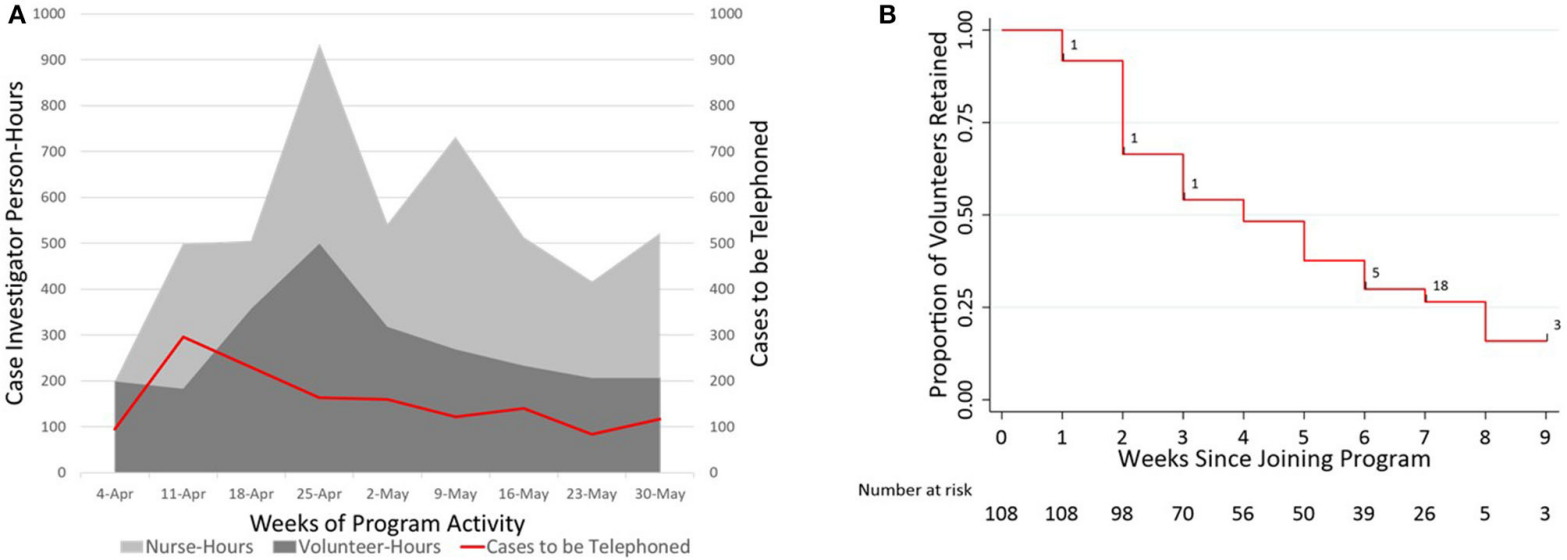
Plots showing supply and demand, and retention of the case investigation workforce over time. **(A)** Contour plot comparing the supply of case investigator time (in person-hours, left axis, volunteers and nurses stacked) to the demand for case investigation (in cases assigned to be telephoned per week, right axis) for each calendar week of program activity. Assuming (conservatively) that an average of 1 h is required to perform and document case investigation (29), the supply of volunteer case investigator time exceeded demand for case investigation in all weeks except the week beginning 11-Apr, when 40 public health nurses were first recruited. **(B)** Retention of case-investigation volunteers (*n* = 108) over time, shown using a survival plot against time from joining until the outcome of leaving the New Haven contact tracing program. Right censoring is noted with black hash marks overlaid on the survival curve, with the corresponding *n*.

The supply of available case investigators exceeded demand for case investigation in all weeks (Figure 3A; contact notifiers presented in Supplementary Figure 3), although it was necessary to add public health nurses during the program's second week to meet demand. Case investigation volunteers offered a median of 4 h during their first week and decreased involvement by 0.68 h per calendar week in the program (95%CI −0.84 to −0.51, *p* < 0.0001; Supplementary Table 4), with a median time of 4 weeks (95% CI 3–5; Figure 3B) from signing-up for to retiring from the program.

## Discussion

This systematic and structured evaluation of the core processes involved in COVID-19 contact tracing enabled us to quantify the uptake and efficiency of implementation and identify factors influencing its delivery. In this prospective evaluation, we found that low yield and timeliness metrics were closely linked to delays in test reporting and data transfer, incomplete or incorrect outreach information, and limited success in reaching cases and contacts by telephone. We also identified case, contact, and programmatic factors associated with success. Last, we observed high rates of adoption of contact tracing among volunteers, but also high rates of turnover. Below, we use the RE-AIM framework to contextualize our findings and propose potential solutions to improve the delivery of contact tracing for current and future pandemics (Table 2).

**Table 2:** Potential solutions for the identified challenges to contact tracing, by RE-AIM dimension.

**RE-AIM dimension**	**Challenges**	**Potential solutions**
Reach	Lack of required outreach data	Collect case phone numbers and initiate linkage to contact tracing at time-of-testing. Identify messaging strategies (e.g., education regarding importance of contact tracing, security of data, and benefits of contact tracing to one's community, etc.,) to increase completion of evaluation.
	Lower outreach success among the elderly	Prioritize outreach calls to those most at risk and tailor engagement strategies to client needs and preferences.
	Unmeasured characteristics of tracers and cases that influence success in contact outreach	Identify characteristics of tracers, tracer-case dyads, and case social networks that influence success in order to improve and standardize training and outreach strategies. Evaluate strategies for engaging cases in linking contacts to the health department without infringing on privacy or promoting stigma (e.g., training cases to notify their contacts of exposure and inform them of incoming calls from the health department).
Implementation	Delays in test reporting and data transfer	Use same-day electronic linkage to (A) share test results from the lab with contact tracing programs and (B) make case and contact assignments to contact tracers.
	Delays between case and contact outreach attempts	Integrate outreach to cases and their household contacts, as is done with household contact investigation for tuberculosis.
Adoption and maintenance	High turnover amongst volunteer contact tracers	Offer financial or educational incentives to increase sustainability of the contact tracing workforce.

In previous reports, the yield of COVID-19 contact tracing varies widely, with interview success rates ranging 33–100% (13, 18–20, 31) and the proportions of cases reporting contacts ranging 7–100% (13, 19, 20, 31). In our study, missing or incorrect information (e.g., names, phone numbers) was the most significant barrier to *Reach*, affecting nearly one-third of cases and over half of reported contacts. This surprising barrier reflects a hesitancy or inability of many cases to provide complete outreach information for their contacts, which should be explored in future studies. It also reflects a failure of independent testing sites to collect case phone numbers at the time-of-testing. In the haste to establish sufficient numbers of testing sites, the opportunity to link this service with downstream contact tracing was overlooked by many. While some states reported similar challenges to obtaining this information (32) early in the pandemic, by the end of the first year of the pandemic some reported near complete capture of accurate phone numbers (33). These improvements reflect the impact of redesigning care processes, and additional insights into contact tracing efficiency may be found in other disease contexts (4). In contact tracing for tuberculosis, for example, outreach information is rarely missing because case investigation is introduced at diagnosis or treatment initiation and contacts are frequently evaluated in-person during household or office visits. Consequently, tracers in multiple settings routinely reach >80% of tuberculosis contacts (34, 35). While large COVID-19 caseloads and limited personal protective equipment made in-person contact tracing infeasible throughout much of pandemic, the practice of introducing contact tracing and verifying outreach preferences at diagnosis (or earlier at the time of testing) could also be adopted for COVID-19.

We additionally found that individual case characteristics strongly influenced outreach success. The lower likelihood of successful outreach to the elderly is concerning given their increased risk of severe disease (36, 37). While the association of all race/ethnicity categories with successful outreach to cases just missed the significance threshold (*p* = 0.054) after adjustment for time and other potentially confounding factors, the statistical power of the analysis may have been limited by the sample size. Nevertheless, our precision estimates comparing Black and Hispanic/Latinx cases consistently excluded the null hypothesis, suggesting that Black cases were significantly less likely to be interviewed. Both older age and non-white race/ethnicity have been associated with more severe disease and higher mortality (36–38), and improving the reach and timeliness of contact tracing may offer opportunities to intervene earlier to improve individual outcomes. Future studies should continue to explore differences in outcomes across population groups, given that pre-existing health inequities have been amplified by the pandemic (38, 39). In particular, while we were only able to evaluate differences in interview and notification outcomes, future studies should also evaluate predictors of successful isolation and quarantine. Future contact tracing programs should also strive to collect comprehensive race/ethnicity data to help identify and address disparities in access to COVID care (38).

Case and tracer characteristics also appeared to influence contact outcomes, with strong correlations between outcomes of contacts reported by the same cases, elicited by the same investigators, or called by the same notifiers. To standardize training of contact tracers and inform best practices, future studies should explore which characteristics and behaviors of these individuals, dyads, or networks influence success. In the area of HIV partner notification (40), for comparison, index cases often prefer to notify and refer their own contacts for evaluation, an approach that could also be considered for COVID-19.

In terms of *Implementation*, slow test reporting and data transfer led to the most significant delays, as reported elsewhere (18–20). Considering the transmissibility of SARS-CoV-2 and the risks of each day of delay, same-day test results, electronic reporting to public health databases, and early outreach could be better prioritized and even incentivized. In addition, case and contact outreach could be integrated so that all household members are notified concurrently rather than sequentially to improve timeliness and uptake among contacts.

Elsewhere, the ratio of contact tracers to cases and contacts was found to be associated with timeliness and number of contacts identified (21). In evaluating *Adoption*, we found volunteers to be a feasible, although not sustainable, solution to human resource shortages, given the high turnover among volunteers. We separately conducted focus groups with volunteers, described in detail elsewhere (41), who reported that burnout and transitions in academic roles and schedules likely contributed to decreased volunteer availability. Fortunately, the support of public health nurses bolstered capacity during surges and sustained the program. Further research is needed to identify strategies to improve the *Maintenance* of volunteer-driven programs, such as requesting fixed weekly time commitments and offering academic credit or small stipends to incentivize retention.

Many COVID-19 contact tracing programs, including the one evaluated here, struggled to meet CDC's yield and timeliness benchmarks for effective case and contact outreach (16, 17). While the yield of this program was significantly limited by barriers beyond the control of the NHHD (e.g., missing phone number data from independent testing sites, hesitancy or inability of cases to fully report contacts, etc.,), this program still managed to reach nearly 70% of all actionable cases (those with phone numbers) with a median time of 5 days from reporting. They also managed to reach 82% of all actionable contacts (those with requisite outreach information), of whom nearly 70% were reached within 6 days of their exposure. Given the immense constraints on resources and time to establish the emergency response, these outcomes are commendable, even if falling short of target benchmarks.

It is also important to note that there is value to tracing even when it falls short of such benchmarks. While the modeling studies used to derive target benchmarks consider contact tracing as a stand-alone intervention (16), in practice, it is bundled with other interventions, so that contact tracing serves additional pandemic objectives, including health education and linkage to social support (nutritional, financial, etc.), testing, medical care, and vaccination. Bundling interventions to enhance impact is critical to solve what might be described as the pandemic's “Swiss cheese” problem, in which holes in the clinical and public health response arise at multiple levels, times, and locations to sustain the pandemic (42). Qualitative data collected in parallel with this project and published separately (41) supports this idea that even if contact tracing itself has gaps, it may still contribute to the overall public health response. Limited retrospective data from other settings also suggests that contact tracing may also contribute to improved cumulative outcomes (7), and this important question should be evaluated further in future prospective studies.

This study had several important strengths, including its prospective design and use of detailed participant data to identify challenges to and predictors of each step of the process. It is among the first reports on implementation outcomes of contact tracing for COVID-19 in North America and provides insights into resource allocation and volunteer deployment during the early, crisis stage of the COVID-19 pandemic. Insights from this phase of the pandemic will not only help guide intervention adaptations throughout the subsequent phases of the COVID-19 pandemic, but will also help inform responses to future epidemics and pandemics. Last, New Haven has a high level of racial and ethnic diversity, providing an appropriate setting for understanding inequities in implementation processes and outcomes.

There were also some limitations. First, missing demographic data may have biased our analyses in uncertain ways, but we used multiple imputation to help reduce such biases in our models. Second, we did not capture the reasons for unanswered calls, or for refusals to participate, although the viewpoints and experiences of volunteer contacts are presented in detail elsewhere (41), and separate studies will report the viewpoints of cases and contacts regarding these and other barriers to uptake. Third, we could not evaluate under-reporting of contacts and therefore may have overestimated the proportion of contacts reached. Fourth, we were unable to report on effectiveness outcomes such as the proportion of contacts infected because test availability was extremely limited early in the pandemic. Last, these data were collected during the initial months of the COVID-19 pandemic, but contact tracing strategies and barriers have evolved substantially since that time. Nevertheless, there is still much that can be learned from these findings from the initial phase of the pandemic to improve ongoing and future pandemic response efforts, as many related challenges persist.

In conclusion, in this large public health evaluation of an early, volunteer-driven contact tracing program, we found that yield was significantly reduced by missing case and contact information and that timeliness was limited by slow test reporting and data transfer. Volunteers were a feasible but short-term source of contact tracers, and many case, contact, and program characteristics appeared to influence success. Together, these findings point to opportunities for process redesign to increase the impact of contact tracing, with a focus on integrated data management, engagement of all communities, and better understanding of the positive social influences between cases and contacts.

## Data Availability Statement

The raw data supporting the conclusions of this article will be made available by the authors, without undue reservation.

## Ethics Statement

The studies involving human participants were reviewed and approved by Yale Human Subjects Committee. Written informed consent from the participants' legal guardian/next of kin was not required to participate in this study in accordance with the national legislation and the institutional requirements.

## Author Contributions

TS, BW, LG, LN, MB, and JD contributed to study conceptualization. TS, BW, CS, JG, RH, and MB contributed to data collection and curation. Formal analysis conducted by TS, CS, XZ, DS, and JD. Funding was acquired by JD and the project was administered and supervised by MB and JD. TS and JD participated in the drafting of the original manuscript, tables, and figures. All authors participated in review and editing of the manuscript.

## Conflict of Interest

TS, RH, XZ, LG, and JD report a contract between the Connecticut Department of Public Health and Yale School of Public Health for which they provide analytical support for the state's COVID-19 contact tracing program. The remaining authors declare that the research was conducted in the absence of any commercial or financial relationships that could be construed as a potential conflict of interest.

## Publisher's Note

All claims expressed in this article are solely those of the authors and do not necessarily represent those of their affiliated organizations, or those of the publisher, the editors and the reviewers. Any product that may be evaluated in this article, or claim that may be made by its manufacturer, is not guaranteed or endorsed by the publisher.
