# Supplementary material for: Lessons Learned From COVID-19 Contact Tracing During a Public Health Emergency: A Prospective Implementation Study
Source: Front Public Health. 2021 Aug 20;9:721952. doi: 10.3389/fpubh.2021.721952 (PMC8417826; doi:10.3389/fpubh.2021.721952)
Supplement: Supplementary file 1 [file Data_Sheet_1.docx]

**ONLINE SUPPLEMENT**

**Supplementary Table 1**: Predictors^a^ of case interview completion including all cases telephoned (n=1160)

| **Characteristic** | **Unadjusted** | | | **Adjusted** | | |
| --- | --- | --- | --- | --- | --- | --- |
|  | **RR** | **95% CI** | **P-value** | **aRR**^b^ | **95% CI** | **P-value** |
| **Age, years** |  |  | 0.002^c^ |  |  | **0.012**^c^ |
| <18 | 1.02 | 0.88 – 1.19 |  | 1.00 | 0.86 – 1.16 |  |
| 18-35 (ref) | 1 | - |  | 1 | - |  |
| 36-50 | 1.00 | 0.91 – 1.09 |  | 1.00 | 0.91 – 1.08 |  |
| 51-65 | 0.91 | 0.82 – 1.01 |  | 0.92 | 0.83 – 1.02 |  |
| >65 | 0.71 | 0.59 – 0.85 |  | 0.74 | 0.61 – 0.89 |  |
| Male Sex | 0.97 | 0.90 – 1.04 | 0.378 | 0.96 | 0.89 – 1.04 | 0.302 |
| Race/Ethnicity |  |  | 0.006^c^ |  |  | 0.054^c^ |
| Hispanic/Latinx (ref) | 1 | - |  | 1 | - |  |
| Black/African American | 0.86 | 0.78 – 094 |  | 0.88 | 0.80 – 0.97 |  |
| White | 0.85 | 0.73 – 0.99 |  | 0.89 | 0.77 – 1.03 |  |
| Other | 0.92 | 0.71 – 1.19 |  | 0.93 | 0.72 – 1.20 |  |
| **Week since program start (1-9)** | 0.99 | 0.97 – 1.01 | 0.403 | 0.97 | 0.94 – 0.99 | **0.020** |
| Capacity^d^ | 1.01 | 0.99 – 1.03 | 0.385 | 1.03 | 1.00 – 1.06 | 0.063 |

**Abbreviations**: RR, relative risk; 95% CI, 95% Confidence Interval; ref, Reference Category

**Legend:** ^a^Estimates derived from a multivariable log-binomial GEE model; Intraclass correlation coefficient (ICC) for outreach workers=0.002. ^b^Results are reported after adjusting for all other variables in the table. ^c^P-value for overall significance of categorical variable. ^d^Capacity measured via ratio of weekly available case investigator person-hours to weekly incident cases to be telephoned. Bolded covariates indicate covariates that were statistically significant in reference to p<0.05 following multivariable adjustment.

**Supplementary Table 2**: Predictors^a^ of contact reporting^b^ including all contacts reported (n=2437)

| **Characteristic** | **Unadjusted** | | | **Adjusted** | | |
| --- | --- | --- | --- | --- | --- | --- |
|  | **RR** | **95% CI** | **p-value** | **aRR**^c^ | **95% CI** | **p-value** |
| **Case Age, years** |  |  | <0.001^d^ |  |  | **0.008**^d^ |
| <18 | 1.17 | 0.97 – 1.40 |  | 1.00 | 0.83 – 1.21 |  |
| 18-35 (ref) | 1 | - |  | 1 | - |  |
| 36-50 | 0.83 | 0.73 – 0.93 |  | 0.83 | 0.73 – 0.93 |  |
| 51-65 | 1.09 | 0.96 – 1.23 |  | 1.01 | 0.89 – 1.14 |  |
| >65 | 1.04 | 0.87 – 1.24 |  | 0.86 | 0.71 – 1.04 |  |
| Case Male Sex | 0.98 | 0.90 – 1.08 | 0.721 | 0.95 | 0.86 – 1.04 | 0.272 |
| Case Race/Ethnicity |  |  | 0.725^d^ |  |  | 0.224^d^ |
| Hispanic/Latinx (ref) | 1 | - |  | 1 | - |  |
| Black/African American | 0.94 | 0.84 – 1.06 |  | 0.89 | 0.79 – 1.01 |  |
| White | 1.04 | 0.86 – 1.25 |  | 0.99 | 0.82 – 1.19 |  |
| Other | 0.97 | 0.73 – 1.28 |  | 0.85 | 0.64 – 1.13 |  |
| **Contact Age, years** |  |  | <0.001^d^ |  |  | **<0.001**^d^ |
| <18 | 0.68 | 0.58 – 0.78 |  | 0.63 | 0.54 – 0.72 |  |
| 18-35 (ref) | 1 | - |  | 1 | - | - |
| 36-50 | 1.04 | 0.91 – 1.19 |  | 1.07 | 0.93 – 1.22 |  |
| 51-65 | 1.08 | 0.93 – 1.26 |  | 1.11 | 0.96 – 1.29 |  |
| >65 | 0.99 | 0.80 – 1.21 |  | 1.10 | 0.89 – 1.34 |  |
| Contact Male Sex | 0.91 | 0.82 – 1.00 | 0.054 | 0.94 | 0.85 – 1.04 | 0.201 |
| **Non-Household Contact** | 0.77 | 0.66 – 0.89 | <0.001 | 0.88 | 0.77 – 1.00 | **0.0495** |
| **Contact Relationship to Case** |  |  | <0.001^d^ |  |  | **<0.001**^d^ |
| Family member (ref) | 1 | - |  | 1 | - | - |
| Social contact | 0.82 | 0.70 – 0.96 |  | 0.77 | 0.65 – 0.91 |  |
| Work contact | 0.56 | 0.45 – 0.70 |  | 0.57 | 0.44 – 0.74 |  |
| Week (1 – 9) | 0.98 | 0.95 – 1.02 | 0.441 | 0.97 | 0.93 – 1.01 | 0.109 |
| Capacity^e^ | 1.00 | 0.96 – 1.04 | 0.994 | 1.01 | 0.98 – 1.05 | 0.492 |

**Abbreviations**: RR, relative risk; 95% CI, 95% Confidence Interval; ref, Reference Category

**Legend:** ^a^Estimates derived from a multivariable log-binomial GEE model; Intraclass correlation coefficient (ICC) for cases = 0.45; ICC for outreach workers = 0.21. ^b^Contact reporting defined as successful collection of all required outreach information (name, phone number/email, and date of exposure. ^c^Results are reported after adjusting for all other variables in the table. ^d^P-value for overall significance of categorical variable. ^e^Capacity measured via ratio of weekly available case investigator person-hours to weekly incident cases to be telephoned. Bolded covariates indicate covariates that were statistically significant in reference to p<0.05 following multivariable adjustment.

**Supplementary Table 3**: Predictors^a^ of contact notification including all contacts telephoned (n=840)

| **Characteristic** | **Unadjusted** | | | **Adjusted** | | |
| --- | --- | --- | --- | --- | --- | --- |
|  | **RR** | **95% CI** | **P-value** | **aRR**^b^ | **95% CI** | **P-value** |
| Case Age, years |  |  | 0.320^c^ |  |  | 0.144^c^ |
| <18 | 1.20 | 0.48 – 3.01 |  | 1.01 | 0.47 – 2.14 |  |
| 18-35 (ref) | 1 | - |  | 1 | - |  |
| 36-50 | 0.79 | 0.48 – 1.27 |  | 0.77 | 0.52 – 1.14 |  |
| 51-65 | 0.62 | 0.39 – 1.01 |  | 0.61 | 0.42 – 0.90 |  |
| >65 | 0.72 | 0.35 – 1.46 |  | 0.72 | 0.42 – 1.23 |  |
| Case Male Sex | 1.03 | 0.77 – 1.38 | 0.821 | 1.12 | 0.83 – 1.53 | 0.451 |
| Case Race/Ethnicity |  |  | 0.929^c^ |  |  | 0.416^c^ |
| Hispanic/Latinx (ref) | 1 | - |  | 1 | - |  |
| Black/African American | 1.07 | 0.76 – 1.51 |  | 1.29 | 0.90 – 1.85 |  |
| White | 1.08 | 0.65 – 1.78 |  | 1.14 | 0.69 – 1.87 |  |
| Other | 1.43 | 0.39 – 5.24 |  | 1.98 | 0.54 – 7.26 |  |
| Contact Age, years |  |  | 0.744^c^ |  |  | 0.711^c^ |
| <18 | 0.92 | 0.62 – 1.37 |  | 0.89 | 0.60 – 1.34 |  |
| 18-35 (ref) | 1 | - |  | 1 | - |  |
| 36-50 | 1.21 | 0.78 – 1.89 |  | 1.22 | 0.79 – 1.90 |  |
| 51-65 | 1.12 | 0.68 – 1.82 |  | 1.14 | 0.70 – 1.85 |  |
| >65 | 0.87 | 0.48 – 1.58 |  | 0.95 | 0.52 – 1.74 |  |
| Contact Male Sex | 1.12 | 0.83 – 1.51 | 0.451 | 1.11 | 0.82 – 1.51 | 0.499 |
| Non-Household Contact | 1.06 | 0.69 – 1.63 | 0.795 | 1.29 | 0.79 – 2.11 | 0.311 |
| Contact Relationship to Case |  |  | 0.358^c^ |  |  | 0.118^c^ |
| Family member (ref) | 1 | - |  | 1 | - | - |
| Social contact | 0.69 | 0.43 – 1.11 |  | 0.63 | 0.40 – 1.00 |  |
| Work contact | 0.89 | 0.33 – 2.42 |  | 0.61 | 0.28 – 1.30 |  |
| Week (1 – 9) | 1.13 | 1.03 – 1.25 | 0.011 | 1.07 | 0.97 – 1.18 | 0.155 |
| **Capacity**^d^ | 1.61 | 1.10 – 2.36 | 0.015 | 1.43 | 1.04 – 1.95 | **0.026** |

**Abbreviations**: RR, relative risk; 95% CI, 95% Confidence Interval; ref, Reference Category

**Legend:** ^a^Estimates derived from a multivariable log-binomial GEE model; Intraclass correlation coefficient (ICC) for cases = 0.60; ICC for outreach worker = 0.14. ^b^Results are reported after adjusting for all other variables in the table. ^c^P-value for overall significance of categorical variable. ^d^Capacity measured via ratio of weekly available contact notifier person-hours to weekly incident contacts to be telephoned. Bolded covariates indicate covariates that were statistically significant in reference to p<0.05 following multivariable adjustment.

**Supplementary Table 4**: The effect of time since initial volunteer sign-up on hours volunteered per week^a^

| **Characteristic** | **Unadjusted** | | | **Adjusted** | | |
| --- | --- | --- | --- | --- | --- | --- |
|  | **Hours Volunteered**^b^ **(per week)** | **95% CI** | **P-value** | **Hours Volunteered**^b^ **(per week)** | **95% CI** | **P-value** |
| **Time since initial sign-up (weeks)** | **-0.67** | **-0.83** – **-0.51** | **<0.001** | **-0.68** | **-0.84** – **-0.51** | **<0.001** |
| Calendar time of sign-up |  |  | 0.166^c^ |  |  | 0.060^c^ |
| Week 1 (ref) | 1 | - |  | 1 | - |  |
| Week 2 | -1.95 | -3.58 – -0.31 |  | -2.29 | -3.95 – -0.63 |  |
| Week 3 | 0.03 | -1.49 – 1.54 |  | -0.65 | -2.25 – 0.94 |  |
| Week 4 | -1.38 | -2.94 – 0.18 |  | -2.40 | -4.12 – -0.68 |  |
| During or After Week 5 | 0.90 | -2.81 – 4.61 |  | -0.91 | -4.68 – 2.85 |  |

**Abbreviations**: 95% CI, 95% Confidence Interval; ref, Reference Category

**Legend**: ^a^Estimates derived from a multivariable, longitudinal log-binomial GEE model. ^b^Results are reported after adjusting for all other variables in the table. ^c^P-value for overall significance of categorical variable. Bolded covariates indicate covariates that were statistically significant in reference to p<0.05 following multivariable adjustment.

**Supplemental Figure 1:** Contact Tracing Flow Diagram and Indicator Framework

**
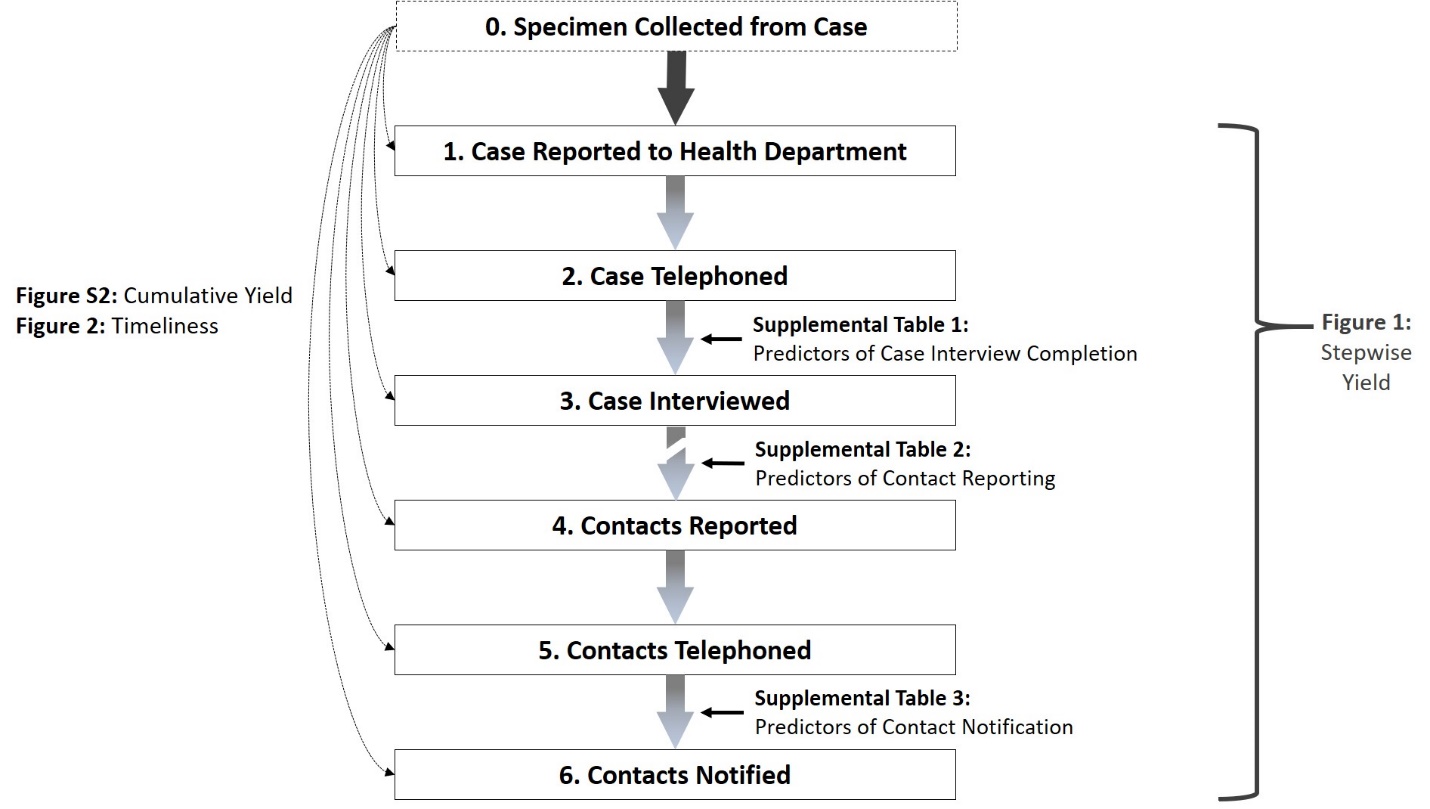
**

**Legend**: This conceptual flow diagram illustrates the framework used to define the six key steps (each numbered) of contact tracing. Stepwise yield (Figure 1) for cases is calculated by dividing the number of cases reaching steps #2-#3 divided by the number that made it to the step prior, while stepwise yield for contacts is calculated by dividing the number of contacts reaching steps #5-#6 divided by the number that made it to the step prior. Indicators for cumulative yield (Supplemental Figure 2) were calculated by dividing the number of cases reaching steps #2-#3 by the total number of cases reported (step #1), and the number of contacts reaching steps #5-#6 divided by the total number of contacts reported (step #4). Indicators for timeliness (Figure 2) were calculated as times between the steps linked by the curved arrows, starting with specimen collection (step #0) and ending with each of the 6 steps (#1-#6). Finally, each of 3 the regression models reported in the Supplemental Tables identify predictors of moving from the previous to the following step**.**

**Supplemental Figure 2**. Cumulative yield indicators for case investigation and contact notification


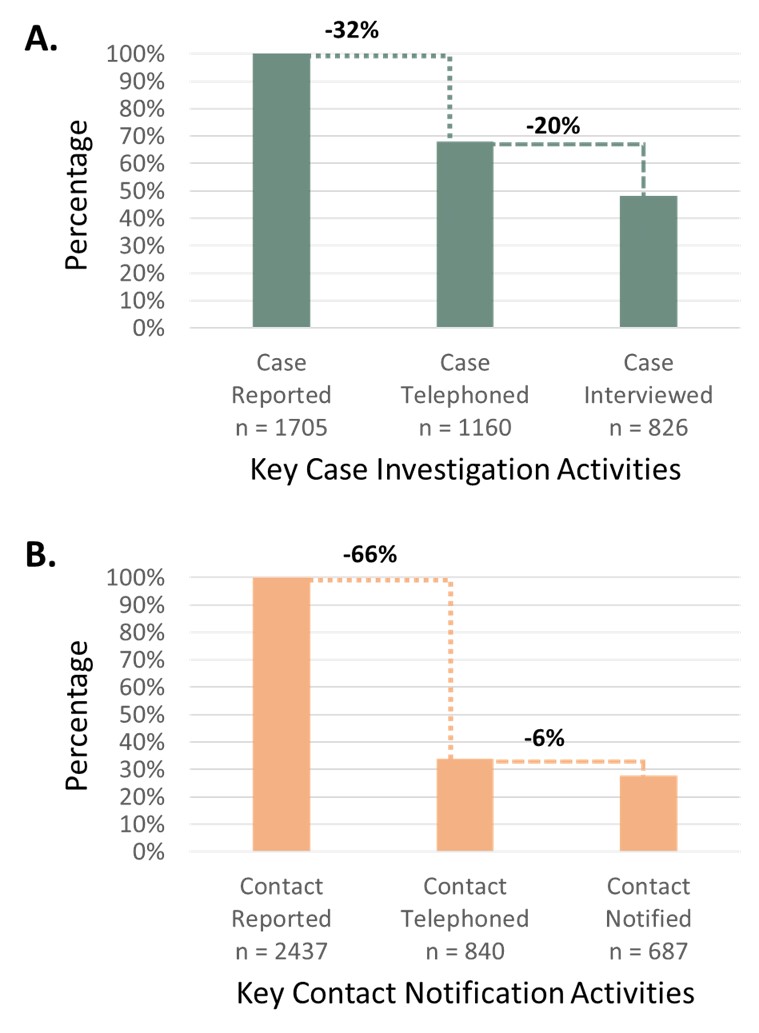


**Legend**: Bar graphs showing the cumulative probability of completing the key contact tracing steps of (A) case investigation and (B) contact notification. Probabilities were calculated by dividing the number completing each step divided by the number reported and multiplying by 100. Dotted lines show the percentages lost between processes, calculated by subtracting the percentage completing the subsequent step from the percentage completing the previous step.

**Supplementary Figure 3**: Plot showing supply of and demand for contact notification volunteers^a^ over time.


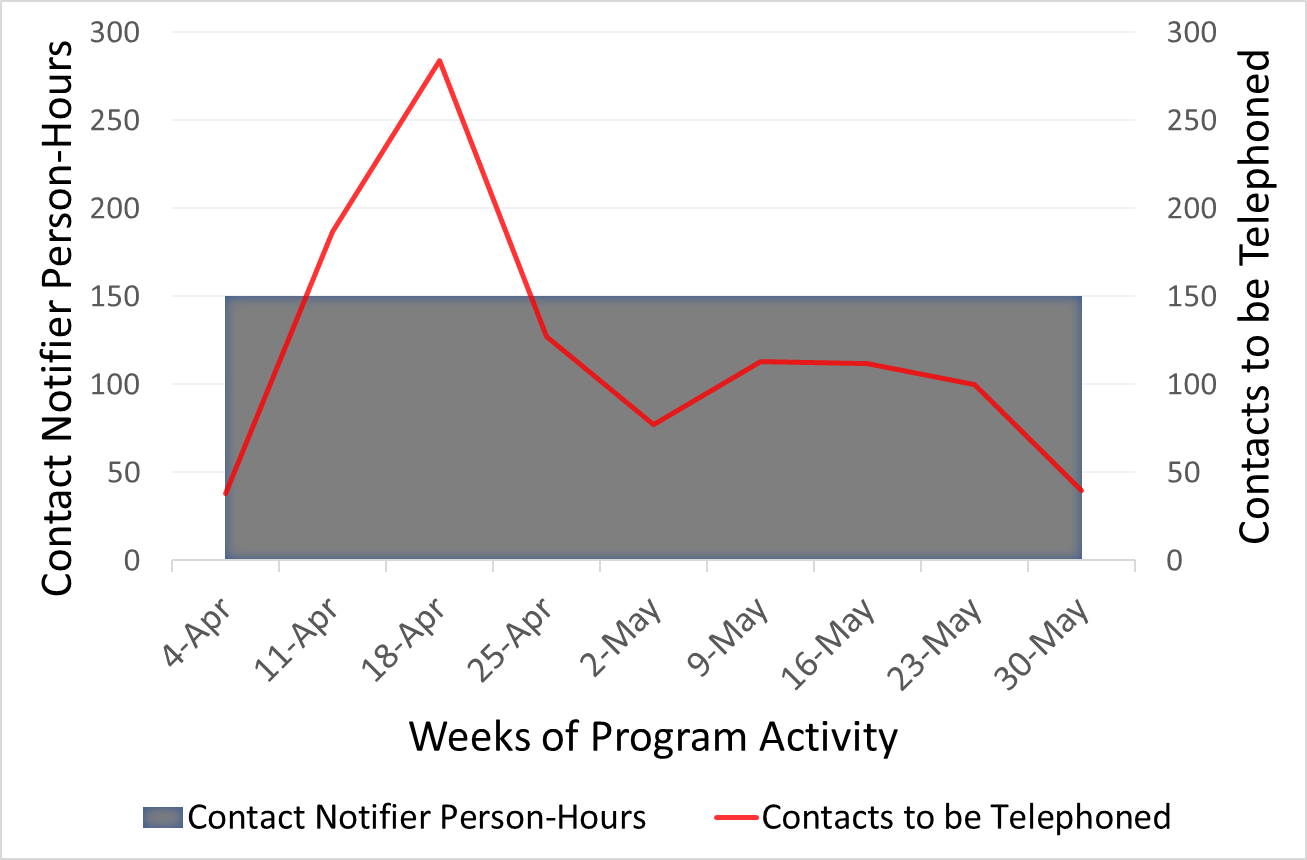


**Legend**: ^a^Contact notification volunteers committed to 5 hours per week, unlike case investigation volunteers who could modify their availability on a weekly basis. Contour plot comparing the supply of contact notifier time (in person-hours, left axis) to the demand for contact notification (in contacts to be telephoned per week, right axis) for each calendar week of program activity. New contacts to be telephoned included those with available outreach information who were within 14 days of last exposure to the case and assigned to a contact tracing volunteer). If we assume, conservatively, that an average of one-half hour is required to perform and document contact notification [29], the supply of contact notifier time exceeded demand for contact notification in all weeks.

**SUPPLEMENTAL METHODS**

*Description of Covariates and Reference Groups for GEE models*

We included age and gender for both cases and contacts, and race/ethnicity for cases only (this information was not collected from contacts) in the first three GEE models evaluating contact tracing outcomes. We also included contact type (household vs. non-household) and relationship (family, social, work). For categorical variables, we selected the largest category as the reference. Age was categorized into the following age groups (ages all in years): <18, 18-35 (reference), 36-50, 51-5, >65. Race categories included: Hispanic/Latinx (reference), Black/African American, White, Other. Contact types included: household (reference) and non-household. Contact relationships included: family (reference), social, and work. We also used non-male sex as a reference category, which included all females except for 2 contacts identified as “other” sex without further specification that were only included in the model predicting collection of outreach information. Because public health nurses shared case assignments, we assigned one random intercept to all cases telephoned by public health nurses.

*Data Missingness*

We determined data to be missing at random based upon (1) lack of identifiable systematic mechanisms leading to data missingness (ruling out *missing not at random*) and (2) observed associations between data missingness and other covariates, as measured via logistic regression (ruling out *missing completely at random)*. Data imputations were performed separately for each model, and the number of imputations was determined by: *(% of missingness) x 100*. The number of imputations for Models 1-3 were 10, 40, and 30, respectively.

**REFERENCES**

1. Blumenthal D, Fowler EJ, Abrams M, Collins SR. Covid-19 - Implications for the Health Care System. N Engl J Med. 2020;383(15):1483-8.

2. Watson C, Cicero A, Blumenstock J, Fraser M. A National Plan to Enable Comprehensive COVID-19 Case Finding and Contact Tracing in the US. The Johns Hopkins Center for Health Security. 2020.

3. Mahachi N, Muchedzi A, Tafuma TA, Mawora P, Kariuki L, Semo BW, et al. Sustained high HIV case-finding through index testing and partner notification services: experiences from three provinces in Zimbabwe. J Int AIDS Soc. 2019;22 Suppl 3:e25321.

4. Hopewell PC, Reichman LB, Castro KG. Parallels and Mutual Lessons in Tuberculosis and COVID-19 Transmission, Prevention, and Control. Emerg Infect Dis. 2021;27(3):681-6.

5. Ooi PL, Lim S, Chew SK. Use of quarantine in the control of SARS in Singapore. Am J Infect Control. 2005;33(5):252-7.

6. Lim PL. Middle East respiratory syndrome (MERS) in Asia: lessons gleaned from the South Korean outbreak. Trans R Soc Trop Med Hyg. 2015;109(9):541-2.

7. Pan A, Liu L, Wang C, Guo H, Hao X, Wang Q, et al. Association of Public Health Interventions With the Epidemiology of the COVID-19 Outbreak in Wuhan, China. JAMA. 2020;323(19):1915-23.

8. Cheng HY, Jian SW, Liu DP, Ng TC, Huang WT, Lin HH, et al. Contact Tracing Assessment of COVID-19 Transmission Dynamics in Taiwan and Risk at Different Exposure Periods Before and After Symptom Onset. JAMA Intern Med. 2020;180(9):1156-63.

9. Fetzer T, Graeber T. Does Contact Tracing Work? Quasi-Experimental Evidence from an Excel Error in England. Working Paper No. 521. 2020.

10. Yalaman A, Basbug G, Elgin C, Galvani AP. Cross-country evidence on the association between contact tracing and COVID-19 case fatality rates. Sci Rep. 2021;11(1):2145.

11. Valent F, Gallo T, Mazzolini E, Pipan C, Sartor A, Merelli M, et al. A cluster of COVID-19 cases in a small Italian town: a successful example of contact tracing and swab collection. Clin Microbiol Infect. 2020;26(8):1112-4.

12. Jian SW, Cheng HY, Huang XT, Liu DP. Contact tracing with digital assistance in Taiwan's COVID-19 outbreak response. Int J Infect Dis. 2020;101:348-52.

13. Lash RR, Moonan PK, Byers BL, Bonacci RA, Bonner KE, Donahue M, et al. COVID-19 Case Investigation and Contact Tracing in the US, 2020. JAMA Network Open. 2021;4(6):e2115850-e.

14. Malheiro R, Figueiredo AL, Magalhaes JP, Teixeira P, Moita I, Moutinho MC, et al. Effectiveness of contact tracing and quarantine on reducing COVID-19 transmission: a retrospective cohort study. Public Health. 2020;189:54-9.

15. Clark E, Chiao EY, Amirian ES. Why contact tracing efforts have failed to curb COVID-19 transmission in much of the U.S. Clin Infect Dis. 2020.

16. Ferretti L, Wymant C, Kendall M, Zhao L, Nurtay A, Abeler-Dorner L, et al. Quantifying SARS-CoV-2 transmission suggests epidemic control with digital contact tracing. Science. 2020;368(6491).

17. Prioritizing COVID-19 Contact Tracing Mathematical Modeling Methods and Findings 2020 [March 29, 2021]. Available from: <https://www.cdc.gov/coronavirus/2019-ncov/php/contact-tracing/contact-tracing-plan/prioritization/mathematicalmodeling.html>.

18. Koetter P, Pelton M, Gonzalo J, Du P, Exten C, Bogale K, et al. Implementation and Process of a COVID-19 Contact Tracing Initiative: Leveraging Health Professional Students to Extend the Workforce During a Pandemic. Am J Infect Control. 2020.

19. Lash RR, Donovan CV, Fleischauer AT, Moore ZS, Harris G, Hayes S, et al. COVID-19 Contact Tracing in Two Counties - North Carolina, June-July 2020. MMWR Morb Mortal Wkly Rep. 2020;69(38):1360-3.

20. Sachdev DD, Brosnan HK, Reid MJA, Kirian M, Cohen SE, Nguyen TQ, et al. Outcomes of Contact Tracing in San Francisco, California—Test and Trace During Shelter-in-Place. JAMA Internal Medicine. 2021;181(3):381-3.

21. Spencer KD, Chung CL, Stargel A, Shultz A, Thorpe PG, Carter MW, et al. COVID-19 Case Investigation and Contact Tracing Efforts from Health Departments - United States, June 25-July 24, 2020. MMWR Morb Mortal Wkly Rep. 2021;70(3):83-7.

22. State of COVID-19 Contact Tracing in the U.S. 2020 [March 29, 2021]. Available from: <https://unitedstatesofcare.org/covid-19/covid-19-contact-tracing/>.

23. Niccolai L, Shelby T, Weeks B, Schenck C, Goodwin J, Hennein R, et al. Community Trace: Rapid Establishment of a Volunteer Contact Tracing Program for COVID-19. Am J Public Health. 2020:e1-e4.

24. Prioritizing COVID-19 Contact Tracing Mathematical Modeling Methods and Findings. Centers for Disease Control and Prevention (CDC). 2020.

25. Hubbard AE, Ahern J, Fleischer NL, Van der Laan M, Lippman SA, Jewell N, et al. To GEE or not to GEE: comparing population average and mixed models for estimating the associations between neighborhood risk factors and health. Epidemiology. 2010;21(4):467-74.

26. Peduzzi P, Concato J, Kemper E, Holford TR, Feinstein AR. A simulation study of the number of events per variable in logistic regression analysis. J Clin Epidemiol. 1996;49(12):1373-9.

27. Jakobsen JC, Gluud C, Wetterslev J, Winkel P. When and how should multiple imputation be used for handling missing data in randomised clinical trials – a practical guide with flowcharts. BMC Medical Research Methodology. 2017;17(1):162.

28. Wu S, Crespi CM, Wong WK. Comparison of methods for estimating the intraclass correlation coefficient for binary responses in cancer prevention cluster randomized trials. Contemp Clin Trials. 2012;33(5):869-80.

29. Case Investigations and Contact Tracing - Frequently Asked Questions 2020 [March 29, 2021]. Available from: <https://www.doh.wa.gov/Emergencies/COVID19/CaseInvestigationsandContactTracing/CaseInvestigationsandContactTracingFAQ>.

30. Glasgow RE, Harden SM, Gaglio B, Rabin B, Smith ML, Porter GC, et al. RE-AIM Planning and Evaluation Framework: Adapting to New Science and Practice With a 20-Year Review. Front Public Health. 2019;7:64.

31. Lash RR, Moonan PK, Byers BL, Bonacci RA, Bonner KE, Donahue M, et al. COVID-19 Case Investigation and Contact Tracing in the US, 2020. JAMA Netw Open. 2021;4(6):e2115850.

32. Miller JS, Bonacci RA, Lash RR, Moonan PK, Houck P, Van Meter JJ, et al. COVID-19 Case Investigation and Contact Tracing in Central Washington State, June-July 2020. J Community Health. 2021.

33. State of Delaware: Coronavirus (COVID-19) Data Dashboard 2021 [March 29, 2021]. Available from: <https://myhealthycommunity.dhss.delaware.gov/locations/state/coronavirus-mitigation#contact_tracing>.

34. New Jersey COVID-19 Dashboard 2021 [March 29, 2021]. Available from: <https://www.nj.gov/health/cd/topics/covid2019_dashboard.shtml>.

35. Jereb J, Etkind SC, Joglar OT, Moore M, Taylor Z. Tuberculosis contact investigations: outcomes in selected areas of the United States, 1999. Int J Tuberc Lung Dis. 2003;7(12 Suppl 3):S384-90.

36. Cavany SM, Sumner T, Vynnycky E, Flach C, White RG, Thomas HL, et al. An evaluation of tuberculosis contact investigations against national standards. Thorax. 2017;72(8):736-45.

37. Cummings MJ, Baldwin MR, Abrams D, Jacobson SD, Meyer BJ, Balough EM, et al. Epidemiology, clinical course, and outcomes of critically ill adults with COVID-19 in New York City: a prospective cohort study. Lancet. 2020;395(10239):1763-70.

38. Ioannou GN, Locke E, Green P, Berry K, O'Hare AM, Shah JA, et al. Risk Factors for Hospitalization, Mechanical Ventilation, or Death Among 10131 US Veterans With SARS-CoV-2 Infection. JAMA Netw Open. 2020;3(9):e2022310.

39. Munoz-Price LS, Nattinger AB, Rivera F, Hanson R, Gmehlin CG, Perez A, et al. Racial Disparities in Incidence and Outcomes Among Patients With COVID-19. JAMA Netw Open. 2020;3(9):e2021892.

40. Berkowitz SA, Cene CW, Chatterjee A. Covid-19 and Health Equity - Time to Think Big. N Engl J Med. 2020;383(12):e76.

41. Kahabuka C, Plotkin M, Christensen A, Brown C, Njozi M, Kisendi R, et al. Addressing the First 90: A Highly Effective Partner Notification Approach Reaches Previously Undiagnosed Sexual Partners in Tanzania. AIDS Behav. 2017;21(8):2551-60.

42. Shelby T, Hennein R, Schenck C, Clark K, Meyer AJ, Goodwin J, et al. Implementation of a volunteer contact tracing program for COVID-19 in the United States: A qualitative focus group study. PLOS ONE. 2021;16(5):e0251033.

43. Reason J. Human error: models and management. BMJ. 2000;320(7237):768-70.

44. Tyler Shelby RH, Christopher Schenck, Katie Clark, Amanda J. Meyer, Justin Goodwin, Brian Weeks, Maritza Bond, Linda Niccolai, J. Lucian Davis and Lauretta E. Grau. Implementation of a Volunteer Contact Tracing Program for COVID-19 in the United States: A Qualitative Focus Group Study. PLOS One, Submitted and in Peer-Review 2021.
